# 2-Ferrocenyl-*N*-(2-ferrocenylbenzo­yl)-*N*-(4-methyl-2-pyrid­yl)benzamide

**DOI:** 10.1107/S1600536809006278

**Published:** 2009-02-28

**Authors:** John F. Gallagher, Steven Alley, Alan J. Lough

**Affiliations:** a School of Chemical Sciences, Dublin City University, Dublin 9, Ireland; b Department of Chemistry, 80 St. George Street, University of Toronto, Toronto, Ontario, Canada M5S 3H6

## Abstract

The title compound, [Fe_2_(C_5_H_5_)_2_(C_30_H_22_N_2_O_2_)], a 2:1 product of the reaction of 2-ferrocenylbenzoic acid and 2-amino-4-methyl­pyridine, forms a twisted mol­ecular structure in the solid state due to steric effects from the two benzene rings *ortho*-substituted with ferrocenyl and carbonyl-derived groups. A short intra­molecular C—H⋯π interaction is observed involving a substituted η^5^-C_5_H_4_ ring and an *ortho* H atom of the benzene ring on the opposite side of the mol­ecule. In the crystal structure, there are no classical hydrogen bonds: inter­actions comprise a short C_6_—H⋯π(C_6_) inter­action involving substituted benzene rings and two C—H⋯O=C inter­actions per mol­ecule.

## Related literature

For background information, see: Gallagher *et al.* (2008 ▶, 2009*a* ▶,*b* ▶). For the parent compound, 2-(dibenzoyl­amino)pyridine, see: Weng *et al.* (2006 ▶). For a related ferrocene derivative, see: Moriuchi & Hirao (2007 ▶). For related structures, see: Akinboye *et al.* (2009*a* ▶,*b* ▶). For a description of the Cambridge Structural Database, see: Allen (2002 ▶).
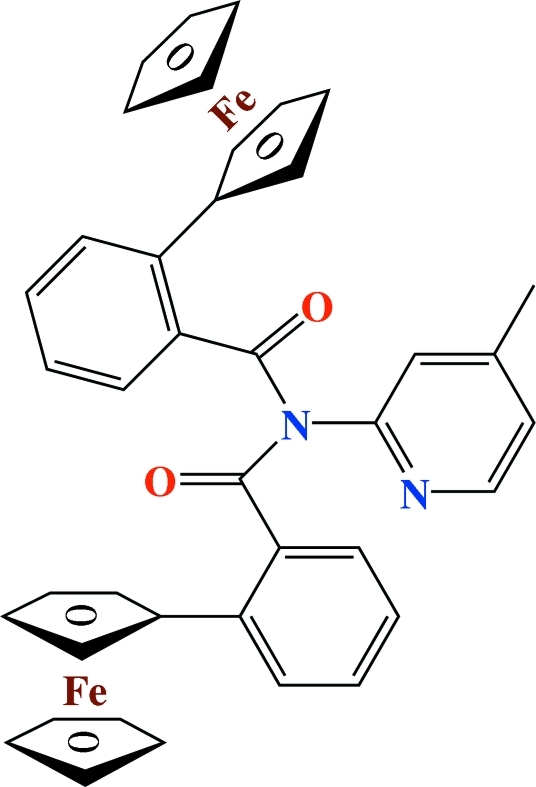


## Experimental

### 

#### Crystal data

[Fe_2_(C_5_H_5_)_2_(C_30_H_22_N_2_O_2_)]
*M* *_r_* = 684.38
Triclinic, 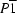

*a* = 12.0861 (7) Å
*b* = 12.4935 (8) Å
*c* = 12.5028 (8) Å
α = 65.159 (3)°
β = 62.696 (3)°
γ = 81.580 (3)°
*V* = 1519.83 (16) Å^3^
*Z* = 2
Mo *K*α radiation
μ = 1.00 mm^−1^
*T* = 150 K
0.22 × 0.22 × 0.14 mm

#### Data collection

Nonius KappaCCD diffractometer
Absorption correction: multi-scan (*SORTAV*; Blessing, 1995 ▶) *T*_min_ = 0.811, *T*_max_ = 0.873
10819 measured reflections
6865 independent reflections
3530 reflections with *I* > 2σ(*I*)
*R*_int_ = 0.107

#### Refinement

*R*[*F* ^2^ > 2σ(*F* ^2^)] = 0.060
*wR*(*F* ^2^) = 0.154
*S* = 0.95
6865 reflections
417 parameters
H-atom parameters constrained
Δρ_max_ = 0.77 e Å^−3^
Δρ_min_ = −0.87 e Å^−3^

### 

Data collection: *KappaCCD Server Software* (Nonius, 1997 ▶); cell refinement: *DENZO-SMN* (Otwinowski & Minor, 1997 ▶); data reduction: *DENZO-SMN*; program(s) used to solve structure: *SHELXS97* (Sheldrick, 2008 ▶); program(s) used to refine structure: *SHELXL97* (Sheldrick, 2008 ▶) and *SORTX* (McArdle, 1995 ▶); molecular graphics: *PLATON* (Spek, 2009 ▶); software used to prepare material for publication: *SHELXL97* and *PREP8* (Ferguson, 1998 ▶).

## Supplementary Material

Crystal structure: contains datablocks global, I. DOI: 10.1107/S1600536809006278/bg2237sup1.cif

Structure factors: contains datablocks I. DOI: 10.1107/S1600536809006278/bg2237Isup2.hkl

Additional supplementary materials:  crystallographic information; 3D view; checkCIF report

**Fig. 2.**
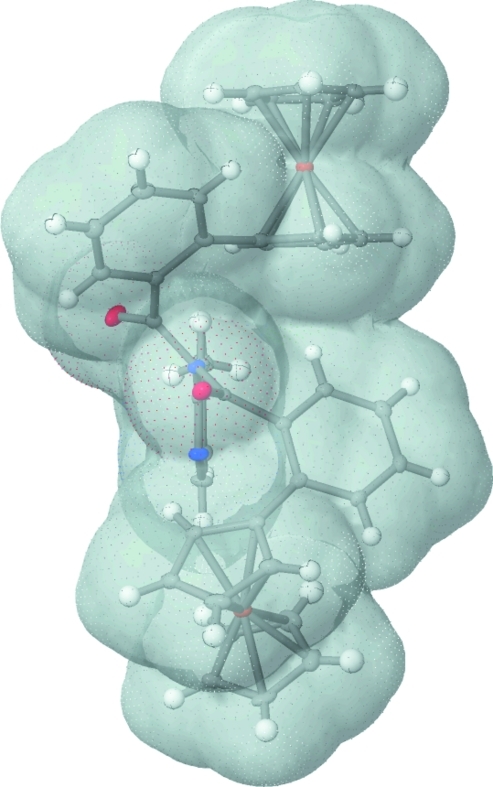
A view of (I) with atoms displayed at the 30% probability level with the intramolecular C—H···π interaction (top centre) and enveloped with an azure coloured van der Waals surface.

Enhanced figure: interactive version of Fig. 2

## Figures and Tables

**Table 1:** Hydrogen-bond geometry (Å, °)

*D*—H⋯*A*	*D*—H	H⋯*A*	*D*⋯*A*	*D*—H⋯*A*
C9—H9*B*⋯O1^i^	0.98	2.36	3.188 (6)	142
C13*B*—H13*B*⋯O2^ii^	0.95	2.58	3.510 (6)	167
C33*B*—H33*B*⋯*Cg*1	0.95	2.58	3.387 (5)	144
C35*A*—H35*A*⋯*Cg*2^iii^	0.95	2.61	3.489 (6)	154

Symmetry codes: (i) 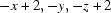; (ii) 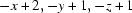; (iii) 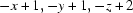. *Cg*1 and *Cg*2 are the centroids of the C11*A*–C15*A*, C31*B*–C36*B* rings, respectively.

**Fig. 1.**
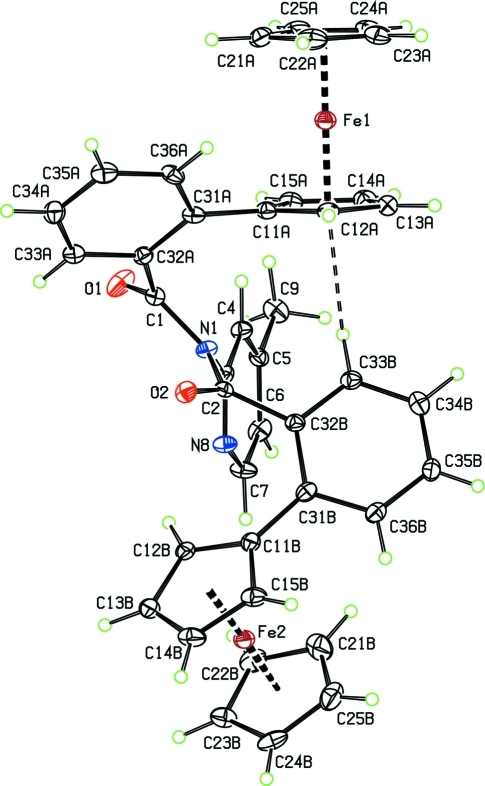
A view of (I) with the atomic numbering scheme. Displacement ellipsoids are drawn at the 30% probability level. The intramolecular C—H···η(C5H4) interaction is depicted with a dashed line.

## supplementary crystallographic information

### Comment

In the condensation reactions of benzoyl chlorides and amino derivatives containing an *ortho*-aromatic ring N atom (such as *ortho*-aminopyridine), two products can be isolated as either the 1:1 or 2:1 benzoyl:pyridine components and with yields and ratios depending on the reaction conditions. We have reported on the structure of the 1:1 derivative namely 2,3-difluoro-*N*-(2-pyridyl)benzamide (Gallagher *et al.*, 2008) and two examples of a 2:1 relative namely the mono-fluoro derivatives, 2-Fluoro-*N*-(2-fluorobenzoyl)-*N*-(2-pyridyl)benzamide and 3-Fluoro-*N*-(3-fluorobenzoyl)-*N*-(2-pyridyl)benzamide (Gallagher *et al.*, 2009*a*,b). Herein, we report an organometallic analogue of these systems where a sterically bulky ferrocenyl group has replaced the fluoro and a different pyridine ring system is used in the molecular scaffold, namely the electroactive molecular system 2-Ferrocenyl-*N*-(2-ferrocenylbenzoyl)-*N*-(4-methyl-2-pyridyl)benzamide (I).

The parent compound 2-(Dibenzoylamino)pyridine has been reported previously (Weng *et al.*, 2006). A review of the literature suggests that structures of this type are rare despite the large number of substituted benzamides reported in the literature. Recently, the crystal structures of two compounds *N*-(3-Br-1,4-dioxo-1,4-dihydro-2-naphthyl)-2-Cl—*N*-(2-chlorobenzoyl)benzamide & *N*-(3-Br-1,4-dioxo-1,4-dihydro-2-naphthyl)-4-*F*—*N*-(4-fluorobenzoyl)benzamide have been reported (Akinboye *et al.*, 2009*a*,b), but these differ substantially from (I) in the quinone scaffold or more specifically in the chloro-1,4-naphthoquinone skeleton. The molecular structure of a related compound which has been examined for protonation-controlled regulation of electronic communication, namely, *N*-Ferrocenecarbonyl-*N*-(2-pyridyl)ferrocenecarboxamide (Moriuchi & Hirao, 2007) is of interest for comparisons with (I) and also the parent compound (Weng *et al.*, 2006).

(I) crystallizes in the triclinic system in space group *P*1 (No. 2) and forms an interesting twisted molecular structure in the solid state due to the steric demands of two *ortho*-substituted benzene rings (Figs. 1–2).

A short intramolecular hydrogen bond is present in (I) involving a substituted η^5^-(C_5_H_4_) ring and an *ortho*-H atom of the benzene ring on the opposite side of the molecule. The geometric data are H33B···*Cg*1 of 2.58 Å, C33B—H33B···*Cg*1 144° and C33B···*Cg*1 3.387 (5) Å (Cg1 is the centroid of the C11A-C15A ring).  This is quite short and comparable to intramolecular _ar_C—H···η^5^-(C_5_) ring interactions present in ferrocene-derived crystal structures deposited on the CSD (Allen, 2002). However, in the vast majority of crystal structures analysed (for such intramolecular interactions) the C—H is only 4/5 bonds from the C_5_ ring and oriented on steric grounds to overlap with the cp ring whether this is energetically favourable or not. The intramolecular interaction in (I) is such that the C—H and substituted cyclopentadienyl ring can be considered to be 8 bonds from each other. However, given the steric demands within (I) a similar situation exists whereby the C—H is oriented towards the ring on steric grounds.

The crystal structure also contains three intermolecular interactions comprising a short C—H···π-(C_6_) interaction {C_6_ is the C31B-C36B ring system, with centroid Cg2} and two C—H···O=C weak interactions per molecule. The former is centrosymmetric and has the effect of (I) joining to form a dimer: the other interactions are less significant in terms of crystal structure packing.

### Experimental

Compound (I) was synthesized *via* standard condensation procedures and similar to the related syntheses reported (Gallagher *et al.*, 2009*a*,b). Separation of the 1:1 and 2:1 derivatives was undertaken by using flash chromatography using CHCl_3_:ethyl acetate. Typical organic workup and washing gave the product (I) in modest yield of 30–40% as a 2:1 component of the mixture. Crystals suitable for X-ray diffraction were grown from CHCl_3_ as colourless blocks over a period of 1–2 weeks. The compounds gave clean ^1^H and ^13^C NMR spectra in CDCl_3_ and infrared spectra (in CHCl_3_ solution, and as KBr disks).

### Refinement

Molecule (I) crystallized in the triclinic system; space group *P*1 (No. 2) and confirmed by the analysis.

H atoms attached to C atoms were treated as riding using the *SHELXL97* (Sheldrick, 2008) defaults at 150 (1) K with C—H = 0.95 Å (aromatic) and 0.98 Å (CH_3_) and *U*_iso_(H) = 1.2*U*_eq_(C) (aromatic) and 1.5*U*_eq_(CH_3_).

### Crystal data

**Table:**

[Fe_2_(C_5_H_5_)_2_(C_30_H_22_N_2_O_2_)]	*Z* = 2
*M**_r_* = 684.38	*F*(000) = 708
Triclinic, *P*1	*D*_x_ = 1.495 Mg m^−^^3^
Hall symbol: -P 1	Mo *K*α radiation, λ = 0.71073 Å
*a* = 12.0861 (7) Å	Cell parameters from 6725 reflections
*b* = 12.4935 (8) Å	θ = 2.6–27.5°
*c* = 12.5028 (8) Å	µ = 1.00 mm^−^^1^
α = 65.159 (3)°	*T* = 150 K
β = 62.696 (3)°	Block, red
γ = 81.580 (3)°	0.22 × 0.22 × 0.14 mm
*V* = 1519.83 (16) Å^3^	

### Data collection

**Table:**

Nonius KappaCCD diffractometer	6865 independent reflections
Radiation source: fine-focus sealed X-ray tube	3530 reflections with *I* > 2σ(*I*)
graphite	*R*_int_ = 0.107
φ scans, and ω scans with κ offsets	θ_max_ = 27.5°, θ_min_ = 2.7°
Absorption correction: multi-scan (*SORTAV*; Blessing, 1995)	*h* = −15→15
*T*_min_ = 0.811, *T*_max_ = 0.873	*k* = −16→13
10819 measured reflections	*l* = −16→16

### Refinement

**Table:**

Refinement on *F*^2^	Secondary atom site location: difference Fourier map
Least-squares matrix: full	Hydrogen site location: inferred from neighbouring sites
*R*[*F*^2^ > 2σ(*F*^2^)] = 0.060	H-atom parameters constrained
*wR*(*F*^2^) = 0.154	*w* = 1/[σ^2^(*F*_o_^2^) + (0.0548*P*)^2^] where *P* = (*F*_o_^2^ + 2*F*_c_^2^)/3
*S* = 0.95	(Δ/σ)_max_ < 0.001
6865 reflections	Δρ_max_ = 0.77 e Å^−^^3^
417 parameters	Δρ_min_ = −0.87 e Å^−^^3^
0 restraints	Extinction correction: *SHELXL97* (Sheldrick, 2008), Fc^*^=kFc[1+0.001xFc^2^λ^3^/sin(2θ)]^-1/4^
Primary atom site location: structure-invariant direct methods	Extinction coefficient: 0.0030 (7)

### Fractional atomic coordinates and isotropic or equivalent isotropic displacement parameters (Å^2^)

**Table:**

	*x*	*y*	*z*	*U*_iso_*/*U*_eq_	
Fe1	0.38802 (6)	0.20628 (6)	1.36143 (6)	0.0257 (2)	
O1	0.8865 (3)	0.2456 (3)	1.0313 (3)	0.0413 (9)	
C1	0.8036 (4)	0.2902 (4)	1.0009 (4)	0.0267 (10)	
N1	0.7691 (3)	0.2461 (3)	0.9319 (3)	0.0222 (8)	
O2	0.7368 (3)	0.4272 (2)	0.7970 (3)	0.0269 (7)	
C2	0.7216 (4)	0.3217 (4)	0.8421 (4)	0.0218 (10)	
C11A	0.5234 (4)	0.2876 (4)	1.1694 (4)	0.0246 (10)	
C12A	0.4007 (4)	0.2944 (4)	1.1760 (4)	0.0258 (10)	
C13A	0.3477 (4)	0.1788 (4)	1.2333 (4)	0.0298 (11)	
C14A	0.4342 (4)	0.0979 (4)	1.2652 (4)	0.0294 (11)	
C15A	0.5410 (4)	0.1638 (4)	1.2281 (4)	0.0262 (10)	
C21A	0.4104 (5)	0.2759 (4)	1.4716 (4)	0.0334 (11)	
C22A	0.2951 (5)	0.3064 (5)	1.4670 (5)	0.0413 (13)	
C23A	0.2237 (5)	0.2030 (5)	1.5185 (5)	0.0436 (14)	
C24A	0.2935 (5)	0.1042 (4)	1.5574 (4)	0.0431 (13)	
C25A	0.4084 (5)	0.1506 (4)	1.5293 (4)	0.0370 (12)	
C31A	0.6109 (4)	0.3872 (4)	1.1173 (4)	0.0240 (10)	
C32A	0.7384 (4)	0.3898 (4)	1.0371 (4)	0.0218 (10)	
C33A	0.8175 (4)	0.4840 (4)	0.9981 (4)	0.0292 (11)	
C34A	0.7710 (4)	0.5758 (4)	1.0373 (4)	0.0311 (11)	
C35A	0.6437 (4)	0.5756 (4)	1.1144 (4)	0.0315 (11)	
C36A	0.5655 (4)	0.4834 (4)	1.1526 (4)	0.0261 (10)	
C3	0.8172 (4)	0.1348 (4)	0.9253 (4)	0.0220 (10)	
C4	0.8075 (4)	0.0369 (4)	1.0373 (4)	0.0245 (10)	
C5	0.8552 (4)	−0.0678 (4)	1.0271 (4)	0.0234 (10)	
C6	0.9085 (4)	−0.0681 (4)	0.9013 (5)	0.0294 (11)	
C7	0.9117 (4)	0.0326 (4)	0.7980 (4)	0.0320 (11)	
N8	0.8687 (3)	0.1363 (3)	0.8062 (3)	0.0249 (8)	
C9	0.8491 (4)	−0.1758 (4)	1.1449 (5)	0.0343 (12)	
Fe2	0.87164 (6)	0.32188 (6)	0.41312 (6)	0.0268 (2)	
C11B	0.7582 (4)	0.3693 (4)	0.5670 (4)	0.0258 (10)	
C12B	0.8869 (4)	0.3777 (4)	0.5378 (4)	0.0277 (10)	
C13B	0.9534 (5)	0.4581 (4)	0.4067 (4)	0.0344 (12)	
C14B	0.8681 (5)	0.5003 (4)	0.3509 (5)	0.0371 (12)	
C15B	0.7481 (4)	0.4470 (4)	0.4484 (4)	0.0307 (11)	
C21B	0.8314 (5)	0.1487 (4)	0.4675 (5)	0.0463 (14)	
C22B	0.9594 (5)	0.1666 (4)	0.4273 (5)	0.0379 (12)	
C23B	1.0115 (4)	0.2511 (4)	0.2970 (5)	0.0340 (12)	
C24B	0.9152 (5)	0.2837 (4)	0.2551 (5)	0.0388 (13)	
C25B	0.8035 (5)	0.2207 (5)	0.3618 (6)	0.0441 (14)	
C31B	0.6561 (4)	0.2927 (4)	0.6881 (4)	0.0240 (10)	
C32B	0.6445 (4)	0.2603 (4)	0.8159 (4)	0.0215 (10)	
C33B	0.5501 (4)	0.1792 (4)	0.9251 (4)	0.0245 (10)	
C34B	0.4630 (4)	0.1321 (4)	0.9116 (4)	0.0276 (10)	
C35B	0.4702 (4)	0.1654 (4)	0.7884 (4)	0.0275 (10)	
C36B	0.5650 (4)	0.2440 (4)	0.6797 (4)	0.0276 (11)	
H12A	0.3617	0.3651	1.1467	0.031*	
H13A	0.2678	0.1586	1.2479	0.036*	
H14A	0.4225	0.0142	1.3047	0.035*	
H15A	0.6128	0.1315	1.2400	0.031*	
H21A	0.4770	0.3292	1.4418	0.040*	
H22A	0.2702	0.3846	1.4341	0.050*	
H23A	0.1422	0.1990	1.5265	0.052*	
H24A	0.2677	0.0231	1.5949	0.052*	
H25A	0.4734	0.1054	1.5461	0.044*	
H33A	0.9042	0.4847	0.9440	0.035*	
H34A	0.8255	0.6387	1.0118	0.037*	
H35A	0.6109	0.6391	1.1406	0.038*	
H36A	0.4785	0.4849	1.2042	0.031*	
H4	0.7683	0.0417	1.1205	0.029*	
H6	0.9421	−0.1377	0.8883	0.035*	
H7	0.9468	0.0296	0.7141	0.038*	
H9A	0.8143	−0.1564	1.2220	0.052*	
H9B	0.9332	−0.2035	1.1288	0.052*	
H9C	0.7958	−0.2382	1.1609	0.052*	
H12B	0.9218	0.3357	0.5973	0.033*	
H13B	1.0399	0.4802	0.3633	0.041*	
H14B	0.8881	0.5548	0.2632	0.045*	
H15B	0.6738	0.4603	0.4375	0.037*	
H21B	0.7735	0.0967	0.5517	0.056*	
H22B	1.0027	0.1282	0.4792	0.045*	
H23B	1.0958	0.2812	0.2458	0.041*	
H24B	0.9244	0.3382	0.1706	0.047*	
H25B	0.7241	0.2258	0.3622	0.053*	
H33B	0.5455	0.1560	1.0098	0.029*	
H34B	0.3986	0.0772	0.9867	0.033*	
H35B	0.4100	0.1344	0.7784	0.033*	
H36B	0.5684	0.2659	0.5957	0.033*	

### Atomic displacement parameters (Å^2^)

**Table:**

	*U*^11^	*U*^22^	*U*^33^	*U*^12^	*U*^13^	*U*^23^
Fe1	0.0273 (4)	0.0268 (4)	0.0221 (4)	0.0010 (3)	−0.0087 (3)	−0.0113 (3)
O1	0.043 (2)	0.050 (2)	0.062 (2)	0.0242 (18)	−0.037 (2)	−0.041 (2)
C1	0.025 (2)	0.032 (3)	0.023 (2)	−0.003 (2)	−0.007 (2)	−0.013 (2)
N1	0.026 (2)	0.024 (2)	0.0210 (19)	0.0063 (16)	−0.0123 (18)	−0.0124 (16)
O2	0.0359 (19)	0.0180 (17)	0.0278 (17)	−0.0006 (14)	−0.0155 (16)	−0.0077 (14)
C2	0.019 (2)	0.026 (3)	0.017 (2)	−0.002 (2)	−0.002 (2)	−0.011 (2)
C11A	0.031 (3)	0.021 (2)	0.019 (2)	0.000 (2)	−0.008 (2)	−0.009 (2)
C12A	0.025 (2)	0.034 (3)	0.019 (2)	0.004 (2)	−0.012 (2)	−0.010 (2)
C13A	0.028 (3)	0.034 (3)	0.025 (3)	−0.004 (2)	−0.010 (2)	−0.011 (2)
C14A	0.036 (3)	0.026 (3)	0.026 (3)	0.000 (2)	−0.008 (2)	−0.015 (2)
C15A	0.025 (2)	0.027 (3)	0.025 (2)	0.008 (2)	−0.008 (2)	−0.015 (2)
C21A	0.047 (3)	0.031 (3)	0.026 (3)	−0.005 (2)	−0.016 (2)	−0.012 (2)
C22A	0.049 (3)	0.041 (3)	0.036 (3)	0.014 (3)	−0.012 (3)	−0.028 (3)
C23A	0.034 (3)	0.065 (4)	0.030 (3)	0.001 (3)	−0.002 (3)	−0.031 (3)
C24A	0.062 (4)	0.029 (3)	0.021 (3)	−0.014 (3)	−0.002 (3)	−0.008 (2)
C25A	0.050 (3)	0.041 (3)	0.022 (3)	0.003 (3)	−0.020 (3)	−0.010 (2)
C31A	0.029 (3)	0.022 (2)	0.018 (2)	0.005 (2)	−0.009 (2)	−0.009 (2)
C32A	0.026 (2)	0.021 (2)	0.021 (2)	0.0012 (19)	−0.011 (2)	−0.0094 (19)
C33A	0.029 (3)	0.033 (3)	0.026 (3)	0.001 (2)	−0.009 (2)	−0.016 (2)
C34A	0.038 (3)	0.023 (3)	0.031 (3)	−0.003 (2)	−0.014 (2)	−0.010 (2)
C35A	0.040 (3)	0.025 (3)	0.031 (3)	0.012 (2)	−0.016 (3)	−0.016 (2)
C36A	0.025 (2)	0.025 (3)	0.023 (2)	0.004 (2)	−0.003 (2)	−0.012 (2)
C3	0.023 (2)	0.022 (2)	0.028 (3)	0.0018 (19)	−0.013 (2)	−0.015 (2)
C4	0.028 (3)	0.024 (2)	0.023 (2)	0.003 (2)	−0.011 (2)	−0.011 (2)
C5	0.019 (2)	0.028 (3)	0.021 (2)	0.0017 (19)	−0.007 (2)	−0.010 (2)
C6	0.034 (3)	0.024 (2)	0.040 (3)	0.011 (2)	−0.020 (2)	−0.020 (2)
C7	0.037 (3)	0.034 (3)	0.021 (2)	0.007 (2)	−0.007 (2)	−0.016 (2)
N8	0.032 (2)	0.023 (2)	0.020 (2)	0.0079 (17)	−0.0117 (18)	−0.0112 (17)
C9	0.033 (3)	0.029 (3)	0.035 (3)	0.006 (2)	−0.011 (2)	−0.012 (2)
Fe2	0.0320 (4)	0.0261 (4)	0.0212 (4)	−0.0022 (3)	−0.0092 (3)	−0.0104 (3)
C11B	0.031 (3)	0.021 (2)	0.024 (2)	−0.004 (2)	−0.009 (2)	−0.011 (2)
C12B	0.035 (3)	0.027 (3)	0.022 (2)	−0.008 (2)	−0.009 (2)	−0.012 (2)
C13B	0.038 (3)	0.035 (3)	0.026 (3)	−0.012 (2)	−0.005 (2)	−0.015 (2)
C14B	0.060 (4)	0.020 (3)	0.021 (3)	−0.005 (2)	−0.009 (3)	−0.007 (2)
C15B	0.041 (3)	0.026 (3)	0.025 (3)	0.006 (2)	−0.013 (2)	−0.013 (2)
C21B	0.058 (4)	0.023 (3)	0.042 (3)	−0.012 (3)	−0.006 (3)	−0.012 (3)
C22B	0.049 (3)	0.033 (3)	0.038 (3)	0.009 (3)	−0.024 (3)	−0.017 (2)
C23B	0.033 (3)	0.039 (3)	0.026 (3)	0.007 (2)	−0.010 (2)	−0.015 (2)
C24B	0.058 (4)	0.040 (3)	0.038 (3)	0.015 (3)	−0.027 (3)	−0.029 (3)
C25B	0.039 (3)	0.052 (4)	0.062 (4)	0.006 (3)	−0.025 (3)	−0.038 (3)
C31B	0.028 (3)	0.021 (2)	0.028 (2)	0.006 (2)	−0.014 (2)	−0.015 (2)
C32B	0.024 (2)	0.021 (2)	0.020 (2)	0.0021 (19)	−0.008 (2)	−0.0102 (19)
C33B	0.029 (3)	0.022 (2)	0.023 (2)	0.003 (2)	−0.011 (2)	−0.011 (2)
C34B	0.024 (2)	0.021 (2)	0.033 (3)	0.002 (2)	−0.008 (2)	−0.011 (2)
C35B	0.022 (2)	0.030 (3)	0.035 (3)	0.000 (2)	−0.011 (2)	−0.018 (2)
C36B	0.029 (3)	0.031 (3)	0.029 (3)	0.003 (2)	−0.016 (2)	−0.015 (2)

### Geometric parameters (Å, °)

**Table:**

Fe1—C11A	2.075 (4)	Fe2—C25B	2.040 (5)
Fe1—C12A	2.046 (4)	C11B—C12B	1.434 (6)
Fe1—C13A	2.037 (4)	C11B—C15B	1.442 (6)
Fe1—C14A	2.030 (4)	C11B—C31B	1.464 (6)
Fe1—C15A	2.027 (4)	C12B—C13B	1.409 (6)
Fe1—C21A	2.036 (4)	C13B—C14B	1.420 (6)
Fe1—C22A	2.046 (5)	C14B—C15B	1.418 (6)
Fe1—C23A	2.045 (5)	C21B—C22B	1.411 (7)
Fe1—C24A	2.042 (5)	C21B—C25B	1.411 (7)
Fe1—C25A	2.036 (4)	C22B—C23B	1.405 (6)
O1—C1	1.211 (4)	C23B—C24B	1.427 (6)
O2—C2	1.200 (5)	C24B—C25B	1.413 (7)
N1—C1	1.422 (5)	C31B—C32B	1.416 (5)
N1—C2	1.422 (5)	C31B—C36B	1.397 (6)
N1—C3	1.446 (5)	C32B—C33B	1.392 (6)
C1—C32A	1.494 (6)	C33B—C34B	1.383 (6)
C2—C32B	1.504 (6)	C34B—C35B	1.379 (6)
C11A—C12A	1.439 (6)	C35B—C36B	1.380 (6)
C11A—C15A	1.439 (6)	C12A—H12A	0.9500
C11A—C31A	1.471 (6)	C13A—H13A	0.9500
C12A—C13A	1.410 (6)	C14A—H14A	0.9500
C13A—C14A	1.417 (6)	C15A—H15A	0.9500
C14A—C15A	1.413 (6)	C21A—H21A	0.9500
C21A—C22A	1.411 (6)	C22A—H22A	0.9500
C21A—C25A	1.420 (6)	C23A—H23A	0.9500
C22A—C23A	1.395 (7)	C24A—H24A	0.9500
C23A—C24A	1.428 (7)	C25A—H25A	0.9500
C24A—C25A	1.416 (7)	C33A—H33A	0.9500
C31A—C32A	1.397 (6)	C34A—H34A	0.9500
C31A—C36A	1.405 (6)	C35A—H35A	0.9500
C32A—C33A	1.399 (6)	C36A—H36A	0.9500
C33A—C34A	1.379 (6)	C4—H4	0.9500
C34A—C35A	1.391 (6)	C6—H6	0.9500
C35A—C36A	1.375 (6)	C7—H7	0.9500
C3—N8	1.318 (5)	C9—H9A	0.9800
C3—C4	1.386 (5)	C9—H9B	0.9800
C4—C5	1.382 (6)	C9—H9C	0.9800
C5—C6	1.401 (6)	C12B—H12B	0.9500
C5—C9	1.503 (6)	C13B—H13B	0.9500
C6—C7	1.362 (6)	C14B—H14B	0.9500
C7—N8	1.352 (5)	C15B—H15B	0.9500
Fe2—C11B	2.057 (4)	C21B—H21B	0.9500
Fe2—C12B	2.041 (4)	C22B—H22B	0.9500
Fe2—C13B	2.045 (5)	C23B—H23B	0.9500
Fe2—C14B	2.030 (4)	C24B—H24B	0.9500
Fe2—C15B	2.039 (4)	C25B—H25B	0.9500
Fe2—C21B	2.037 (5)	C33B—H33B	0.9500
Fe2—C22B	2.050 (5)	C34B—H34B	0.9500
Fe2—C23B	2.034 (5)	C35B—H35B	0.9500
Fe2—C24B	2.042 (5)	C36B—H36B	0.9500
			
C15A—Fe1—C14A	40.78 (17)	C5—C6—C7	119.2 (4)
C15A—Fe1—C25A	107.92 (19)	N8—C7—C6	124.8 (4)
C14A—Fe1—C25A	118.78 (19)	C3—N8—C7	115.3 (4)
C15A—Fe1—C21A	118.95 (19)	C5—C4—H4	120.2
C14A—Fe1—C21A	153.06 (18)	C3—C4—H4	120.2
C25A—Fe1—C21A	40.83 (18)	C7—C6—H6	120.4
C15A—Fe1—C13A	68.56 (17)	C5—C6—H6	120.4
C14A—Fe1—C13A	40.78 (16)	N8—C7—H7	117.6
C25A—Fe1—C13A	152.84 (19)	C6—C7—H7	117.6
C21A—Fe1—C13A	164.92 (18)	C5—C9—H9A	109.5
C15A—Fe1—C24A	126.79 (19)	C5—C9—H9B	109.5
C14A—Fe1—C24A	107.23 (19)	H9A—C9—H9B	109.5
C25A—Fe1—C24A	40.64 (19)	C5—C9—H9C	109.5
C21A—Fe1—C24A	68.8 (2)	H9A—C9—H9C	109.5
C13A—Fe1—C24A	118.5 (2)	H9B—C9—H9C	109.5
C15A—Fe1—C23A	165.2 (2)	C14B—Fe2—C23B	119.0 (2)
C14A—Fe1—C23A	127.4 (2)	C14B—Fe2—C21B	163.9 (2)
C25A—Fe1—C23A	68.0 (2)	C23B—Fe2—C21B	68.0 (2)
C21A—Fe1—C23A	68.0 (2)	C14B—Fe2—C15B	40.80 (18)
C13A—Fe1—C23A	108.17 (19)	C23B—Fe2—C15B	154.61 (18)
C24A—Fe1—C23A	40.89 (19)	C21B—Fe2—C15B	127.2 (2)
C15A—Fe1—C22A	153.4 (2)	C14B—Fe2—C25B	125.9 (2)
C14A—Fe1—C22A	164.9 (2)	C23B—Fe2—C25B	68.6 (2)
C25A—Fe1—C22A	67.9 (2)	C21B—Fe2—C25B	40.5 (2)
C21A—Fe1—C22A	40.45 (18)	C15B—Fe2—C25B	108.59 (19)
C13A—Fe1—C22A	127.43 (19)	C14B—Fe2—C12B	68.41 (18)
C24A—Fe1—C22A	68.2 (2)	C23B—Fe2—C12B	124.29 (18)
C23A—Fe1—C22A	39.9 (2)	C21B—Fe2—C12B	121.2 (2)
C15A—Fe1—C12A	68.63 (16)	C15B—Fe2—C12B	68.65 (18)
C14A—Fe1—C12A	68.43 (18)	C25B—Fe2—C12B	156.3 (2)
C25A—Fe1—C12A	165.56 (19)	C14B—Fe2—C24B	107.0 (2)
C21A—Fe1—C12A	127.45 (18)	C23B—Fe2—C24B	40.98 (17)
C13A—Fe1—C12A	40.40 (17)	C21B—Fe2—C24B	67.9 (2)
C24A—Fe1—C12A	152.6 (2)	C15B—Fe2—C24B	120.34 (18)
C23A—Fe1—C12A	118.94 (19)	C25B—Fe2—C24B	40.5 (2)
C22A—Fe1—C12A	108.50 (19)	C12B—Fe2—C24B	161.6 (2)
C15A—Fe1—C11A	41.04 (16)	C14B—Fe2—C13B	40.79 (18)
C14A—Fe1—C11A	68.86 (18)	C23B—Fe2—C13B	106.1 (2)
C25A—Fe1—C11A	127.46 (19)	C21B—Fe2—C13B	154.7 (2)
C21A—Fe1—C11A	107.71 (18)	C15B—Fe2—C13B	68.59 (19)
C13A—Fe1—C11A	68.60 (17)	C25B—Fe2—C13B	162.5 (2)
C24A—Fe1—C11A	165.06 (19)	C12B—Fe2—C13B	40.36 (17)
C23A—Fe1—C11A	152.7 (2)	C24B—Fe2—C13B	124.8 (2)
C22A—Fe1—C11A	119.2 (2)	C14B—Fe2—C22B	153.7 (2)
C12A—Fe1—C11A	40.88 (16)	C23B—Fe2—C22B	40.25 (18)
O1—C1—N1	119.3 (4)	C21B—Fe2—C22B	40.37 (19)
O1—C1—C32A	119.8 (4)	C15B—Fe2—C22B	164.1 (2)
N1—C1—C32A	120.9 (3)	C25B—Fe2—C22B	68.2 (2)
C1—N1—C2	120.8 (3)	C12B—Fe2—C22B	107.68 (18)
C1—N1—C3	117.2 (3)	C24B—Fe2—C22B	68.04 (19)
C2—N1—C3	119.5 (3)	C13B—Fe2—C22B	119.3 (2)
O2—C2—N1	121.6 (4)	C14B—Fe2—C11B	69.11 (18)
O2—C2—C32B	123.0 (4)	C23B—Fe2—C11B	162.10 (17)
N1—C2—C32B	115.2 (4)	C21B—Fe2—C11B	108.8 (2)
C15A—C11A—C12A	105.9 (4)	C15B—Fe2—C11B	41.21 (16)
C15A—C11A—C31A	127.2 (4)	C25B—Fe2—C11B	121.3 (2)
C12A—C11A—C31A	126.9 (4)	C12B—Fe2—C11B	40.97 (17)
C15A—C11A—Fe1	67.7 (2)	C24B—Fe2—C11B	155.85 (19)
C12A—C11A—Fe1	68.5 (2)	C13B—Fe2—C11B	68.81 (18)
C31A—C11A—Fe1	126.8 (3)	C22B—Fe2—C11B	126.01 (18)
C13A—C12A—C11A	108.8 (4)	C12B—C11B—C15B	106.2 (4)
C13A—C12A—Fe1	69.5 (2)	C12B—C11B—C31B	128.6 (4)
C11A—C12A—Fe1	70.6 (2)	C15B—C11B—C31B	125.1 (4)
C13A—C12A—H12A	125.6	C12B—C11B—Fe2	68.9 (2)
C11A—C12A—H12A	125.6	C15B—C11B—Fe2	68.7 (2)
Fe1—C12A—H12A	125.9	C31B—C11B—Fe2	124.0 (3)
C12A—C13A—C14A	108.4 (4)	C13B—C12B—C11B	109.2 (4)
C12A—C13A—Fe1	70.2 (2)	C13B—C12B—Fe2	70.0 (2)
C14A—C13A—Fe1	69.3 (2)	C11B—C12B—Fe2	70.1 (2)
C12A—C13A—H13A	125.8	C13B—C12B—H12B	125.4
C14A—C13A—H13A	125.8	C11B—C12B—H12B	125.4
Fe1—C13A—H13A	126.3	Fe2—C12B—H12B	126.1
C15A—C14A—C13A	107.9 (4)	C12B—C13B—C14B	107.9 (4)
C15A—C14A—Fe1	69.5 (2)	C12B—C13B—Fe2	69.7 (2)
C13A—C14A—Fe1	69.9 (3)	C14B—C13B—Fe2	69.0 (3)
C15A—C14A—H14A	126.0	C12B—C13B—H13B	126.0
C13A—C14A—H14A	126.0	C14B—C13B—H13B	126.0
Fe1—C14A—H14A	126.2	Fe2—C13B—H13B	126.9
C14A—C15A—C11A	109.0 (4)	C15B—C14B—C13B	108.3 (4)
C14A—C15A—Fe1	69.7 (2)	C15B—C14B—Fe2	70.0 (3)
C11A—C15A—Fe1	71.3 (2)	C13B—C14B—Fe2	70.2 (3)
C14A—C15A—H15A	125.5	C15B—C14B—H14B	125.8
C11A—C15A—H15A	125.5	C13B—C14B—H14B	125.8
Fe1—C15A—H15A	125.1	Fe2—C14B—H14B	125.6
C22A—C21A—C25A	107.2 (4)	C14B—C15B—C11B	108.3 (4)
C22A—C21A—Fe1	70.1 (3)	C14B—C15B—Fe2	69.2 (3)
C25A—C21A—Fe1	69.6 (3)	C11B—C15B—Fe2	70.1 (2)
C22A—C21A—H21A	126.4	C14B—C15B—H15B	125.9
C25A—C21A—H21A	126.4	C11B—C15B—H15B	125.9
Fe1—C21A—H21A	125.5	Fe2—C15B—H15B	126.4
C23A—C22A—C21A	108.8 (4)	C22B—C21B—C25B	108.7 (5)
C23A—C22A—Fe1	70.0 (3)	C22B—C21B—Fe2	70.3 (3)
C21A—C22A—Fe1	69.4 (3)	C25B—C21B—Fe2	69.9 (3)
C23A—C22A—H22A	125.6	C22B—C21B—H21B	125.6
C21A—C22A—H22A	125.6	C25B—C21B—H21B	125.6
Fe1—C22A—H22A	126.5	Fe2—C21B—H21B	125.8
C22A—C23A—C24A	108.6 (5)	C23B—C22B—C21B	108.0 (4)
C22A—C23A—Fe1	70.1 (3)	C23B—C22B—Fe2	69.3 (3)
C24A—C23A—Fe1	69.4 (3)	C21B—C22B—Fe2	69.3 (3)
C22A—C23A—H23A	125.7	C23B—C22B—H22B	126.0
C24A—C23A—H23A	125.7	C21B—C22B—H22B	126.0
Fe1—C23A—H23A	126.4	Fe2—C22B—H22B	126.9
C25A—C24A—C23A	106.7 (4)	C22B—C23B—C24B	107.9 (4)
C25A—C24A—Fe1	69.5 (3)	C22B—C23B—Fe2	70.5 (3)
C23A—C24A—Fe1	69.7 (3)	C24B—C23B—Fe2	69.8 (3)
C25A—C24A—H24A	126.7	C22B—C23B—H23B	126.1
C23A—C24A—H24A	126.7	C24B—C23B—H23B	126.1
Fe1—C24A—H24A	125.8	Fe2—C23B—H23B	125.2
C24A—C25A—C21A	108.7 (4)	C25B—C24B—C23B	107.9 (4)
C24A—C25A—Fe1	69.9 (3)	C25B—C24B—Fe2	69.7 (3)
C21A—C25A—Fe1	69.6 (2)	C23B—C24B—Fe2	69.2 (3)
C24A—C25A—H25A	125.6	C25B—C24B—H24B	126.1
C21A—C25A—H25A	125.6	C23B—C24B—H24B	126.1
Fe1—C25A—H25A	126.5	Fe2—C24B—H24B	126.7
C32A—C31A—C36A	117.9 (4)	C21B—C25B—C24B	107.6 (4)
C32A—C31A—C11A	123.4 (4)	C21B—C25B—Fe2	69.7 (3)
C36A—C31A—C11A	118.7 (4)	C24B—C25B—Fe2	69.8 (3)
C31A—C32A—C33A	120.2 (4)	C21B—C25B—H25B	126.2
C31A—C32A—C1	124.6 (4)	C24B—C25B—H25B	126.2
C33A—C32A—C1	114.7 (4)	Fe2—C25B—H25B	125.9
C34A—C33A—C32A	120.7 (4)	C36B—C31B—C32B	116.4 (4)
C34A—C33A—H33A	119.6	C36B—C31B—C11B	119.2 (4)
C32A—C33A—H33A	119.6	C32B—C31B—C11B	124.4 (4)
C33A—C34A—C35A	119.5 (4)	C33B—C32B—C31B	120.6 (4)
C33A—C34A—H34A	120.2	C33B—C32B—C2	116.9 (4)
C35A—C34A—H34A	120.2	C31B—C32B—C2	122.1 (4)
C36A—C35A—C34A	120.0 (4)	C34B—C33B—C32B	120.8 (4)
C36A—C35A—H35A	120.0	C34B—C33B—H33B	119.6
C34A—C35A—H35A	120.0	C32B—C33B—H33B	119.6
C35A—C36A—C31A	121.6 (4)	C35B—C34B—C33B	119.5 (4)
C35A—C36A—H36A	119.2	C35B—C34B—H34B	120.2
C31A—C36A—H36A	119.2	C33B—C34B—H34B	120.2
N8—C3—C4	124.5 (4)	C34B—C35B—C36B	119.9 (4)
N8—C3—N1	114.2 (4)	C34B—C35B—H35B	120.1
C4—C3—N1	121.3 (4)	C36B—C35B—H35B	120.1
C3—C4—C5	119.6 (4)	C35B—C36B—C31B	122.7 (4)
C4—C5—C6	116.5 (4)	C35B—C36B—H36B	118.6
C4—C5—C9	121.7 (4)	C31B—C36B—H36B	118.6
C6—C5—C9	121.8 (4)		
			
O1—C1—N1—C2	−149.6 (4)	C2—N1—C3—C4	−148.0 (4)
C32A—C1—N1—C2	30.9 (6)	N8—C3—C4—C5	0.7 (6)
O1—C1—N1—C3	12.4 (6)	N1—C3—C4—C5	−179.6 (4)
C32A—C1—N1—C3	−167.1 (4)	C3—C4—C5—C6	−1.6 (6)
C1—N1—C2—O2	18.5 (6)	C3—C4—C5—C9	178.9 (4)
C3—N1—C2—O2	−143.1 (4)	C4—C5—C6—C7	0.7 (6)
C1—N1—C2—C32B	−157.5 (4)	C9—C5—C6—C7	−179.8 (4)
C3—N1—C2—C32B	40.9 (5)	C5—C6—C7—N8	1.3 (7)
C14A—Fe1—C11A—C15A	−37.6 (2)	C4—C3—N8—C7	1.2 (6)
C25A—Fe1—C11A—C15A	73.1 (3)	N1—C3—N8—C7	−178.5 (4)
C21A—Fe1—C11A—C15A	114.0 (3)	C6—C7—N8—C3	−2.2 (7)
C13A—Fe1—C11A—C15A	−81.5 (3)	C14B—Fe2—C11B—C12B	−80.7 (3)
C24A—Fe1—C11A—C15A	39.9 (8)	C23B—Fe2—C11B—C12B	39.7 (7)
C23A—Fe1—C11A—C15A	−169.8 (4)	C21B—Fe2—C11B—C12B	116.3 (3)
C22A—Fe1—C11A—C15A	156.6 (3)	C15B—Fe2—C11B—C12B	−118.2 (4)
C12A—Fe1—C11A—C15A	−118.6 (4)	C25B—Fe2—C11B—C12B	159.2 (3)
C15A—Fe1—C11A—C12A	118.6 (4)	C24B—Fe2—C11B—C12B	−165.9 (4)
C14A—Fe1—C11A—C12A	81.1 (3)	C13B—Fe2—C11B—C12B	−36.9 (3)
C25A—Fe1—C11A—C12A	−168.2 (3)	C22B—Fe2—C11B—C12B	74.7 (3)
C21A—Fe1—C11A—C12A	−127.3 (3)	C14B—Fe2—C11B—C15B	37.4 (3)
C13A—Fe1—C11A—C12A	37.1 (2)	C23B—Fe2—C11B—C15B	157.8 (6)
C24A—Fe1—C11A—C12A	158.5 (7)	C21B—Fe2—C11B—C15B	−125.5 (3)
C23A—Fe1—C11A—C12A	−51.2 (5)	C25B—Fe2—C11B—C15B	−82.7 (3)
C22A—Fe1—C11A—C12A	−84.8 (3)	C12B—Fe2—C11B—C15B	118.2 (4)
C15A—Fe1—C11A—C31A	−120.6 (4)	C24B—Fe2—C11B—C15B	−47.7 (6)
C14A—Fe1—C11A—C31A	−158.2 (4)	C13B—Fe2—C11B—C15B	81.3 (3)
C25A—Fe1—C11A—C31A	−47.5 (4)	C22B—Fe2—C11B—C15B	−167.2 (3)
C21A—Fe1—C11A—C31A	−6.6 (4)	C14B—Fe2—C11B—C31B	156.2 (4)
C13A—Fe1—C11A—C31A	157.9 (4)	C23B—Fe2—C11B—C31B	−83.4 (7)
C24A—Fe1—C11A—C31A	−80.7 (8)	C21B—Fe2—C11B—C31B	−6.8 (4)
C23A—Fe1—C11A—C31A	69.6 (6)	C15B—Fe2—C11B—C31B	118.8 (5)
C22A—Fe1—C11A—C31A	36.0 (4)	C25B—Fe2—C11B—C31B	36.1 (4)
C12A—Fe1—C11A—C31A	120.7 (4)	C12B—Fe2—C11B—C31B	−123.1 (5)
C15A—C11A—C12A—C13A	−1.6 (5)	C24B—Fe2—C11B—C31B	71.0 (6)
C31A—C11A—C12A—C13A	−179.7 (4)	C13B—Fe2—C11B—C31B	−160.0 (4)
Fe1—C11A—C12A—C13A	−59.1 (3)	C22B—Fe2—C11B—C31B	−48.4 (4)
C15A—C11A—C12A—Fe1	57.6 (3)	C15B—C11B—C12B—C13B	0.4 (5)
C31A—C11A—C12A—Fe1	−120.6 (4)	C31B—C11B—C12B—C13B	176.6 (4)
C15A—Fe1—C12A—C13A	81.6 (3)	Fe2—C11B—C12B—C13B	59.3 (3)
C14A—Fe1—C12A—C13A	37.6 (3)	C15B—C11B—C12B—Fe2	−58.8 (3)
C25A—Fe1—C12A—C13A	160.4 (7)	C31B—C11B—C12B—Fe2	117.3 (5)
C21A—Fe1—C12A—C13A	−167.6 (3)	C14B—Fe2—C12B—C13B	−37.7 (3)
C24A—Fe1—C12A—C13A	−48.3 (5)	C23B—Fe2—C12B—C13B	73.5 (3)
C23A—Fe1—C12A—C13A	−84.3 (3)	C21B—Fe2—C12B—C13B	156.8 (3)
C22A—Fe1—C12A—C13A	−126.6 (3)	C15B—Fe2—C12B—C13B	−81.7 (3)
C11A—Fe1—C12A—C13A	119.8 (4)	C25B—Fe2—C12B—C13B	−169.4 (4)
C15A—Fe1—C12A—C11A	−38.2 (2)	C24B—Fe2—C12B—C13B	41.3 (7)
C14A—Fe1—C12A—C11A	−82.2 (3)	C22B—Fe2—C12B—C13B	114.7 (3)
C25A—Fe1—C12A—C11A	40.6 (8)	C11B—Fe2—C12B—C13B	−120.2 (4)
C21A—Fe1—C12A—C11A	72.6 (3)	C14B—Fe2—C12B—C11B	82.6 (3)
C13A—Fe1—C12A—C11A	−119.8 (4)	C23B—Fe2—C12B—C11B	−166.3 (3)
C24A—Fe1—C12A—C11A	−168.2 (4)	C21B—Fe2—C12B—C11B	−82.9 (3)
C23A—Fe1—C12A—C11A	155.9 (3)	C15B—Fe2—C12B—C11B	38.6 (3)
C22A—Fe1—C12A—C11A	113.6 (3)	C25B—Fe2—C12B—C11B	−49.2 (6)
C11A—C12A—C13A—C14A	0.9 (5)	C24B—Fe2—C12B—C11B	161.5 (5)
Fe1—C12A—C13A—C14A	−59.0 (3)	C13B—Fe2—C12B—C11B	120.2 (4)
C11A—C12A—C13A—Fe1	59.9 (3)	C22B—Fe2—C12B—C11B	−125.0 (3)
C15A—Fe1—C13A—C12A	−81.8 (3)	C11B—C12B—C13B—C14B	−0.8 (5)
C14A—Fe1—C13A—C12A	−119.6 (4)	Fe2—C12B—C13B—C14B	58.6 (3)
C25A—Fe1—C13A—C12A	−169.4 (4)	C11B—C12B—C13B—Fe2	−59.4 (3)
C21A—Fe1—C13A—C12A	41.0 (8)	C14B—Fe2—C13B—C12B	119.6 (4)
C24A—Fe1—C13A—C12A	156.9 (3)	C23B—Fe2—C13B—C12B	−124.5 (3)
C23A—Fe1—C13A—C12A	113.6 (3)	C21B—Fe2—C13B—C12B	−52.0 (6)
C22A—Fe1—C13A—C12A	73.6 (3)	C15B—Fe2—C13B—C12B	81.8 (3)
C11A—Fe1—C13A—C12A	−37.6 (2)	C25B—Fe2—C13B—C12B	165.8 (6)
C15A—Fe1—C13A—C14A	37.8 (3)	C24B—Fe2—C13B—C12B	−165.3 (3)
C25A—Fe1—C13A—C14A	−49.8 (5)	C22B—Fe2—C13B—C12B	−83.0 (3)
C21A—Fe1—C13A—C14A	160.6 (7)	C11B—Fe2—C13B—C12B	37.4 (3)
C24A—Fe1—C13A—C14A	−83.5 (3)	C23B—Fe2—C13B—C14B	115.9 (3)
C23A—Fe1—C13A—C14A	−126.8 (3)	C21B—Fe2—C13B—C14B	−171.6 (4)
C22A—Fe1—C13A—C14A	−166.8 (3)	C15B—Fe2—C13B—C14B	−37.8 (3)
C12A—Fe1—C13A—C14A	119.6 (4)	C25B—Fe2—C13B—C14B	46.2 (8)
C11A—Fe1—C13A—C14A	82.0 (3)	C12B—Fe2—C13B—C14B	−119.6 (4)
C12A—C13A—C14A—C15A	0.2 (5)	C24B—Fe2—C13B—C14B	75.1 (3)
Fe1—C13A—C14A—C15A	−59.3 (3)	C22B—Fe2—C13B—C14B	157.5 (3)
C12A—C13A—C14A—Fe1	59.5 (3)	C11B—Fe2—C13B—C14B	−82.2 (3)
C25A—Fe1—C14A—C15A	−84.3 (3)	C12B—C13B—C14B—C15B	0.8 (5)
C21A—Fe1—C14A—C15A	−49.8 (5)	Fe2—C13B—C14B—C15B	59.8 (3)
C13A—Fe1—C14A—C15A	119.2 (4)	C12B—C13B—C14B—Fe2	−59.0 (3)
C24A—Fe1—C14A—C15A	−126.9 (3)	C23B—Fe2—C14B—C15B	159.8 (3)
C23A—Fe1—C14A—C15A	−167.5 (3)	C21B—Fe2—C14B—C15B	47.8 (8)
C22A—Fe1—C14A—C15A	163.0 (7)	C25B—Fe2—C14B—C15B	76.4 (3)
C12A—Fe1—C14A—C15A	81.9 (3)	C12B—Fe2—C14B—C15B	−81.9 (3)
C11A—Fe1—C14A—C15A	37.8 (2)	C24B—Fe2—C14B—C15B	117.0 (3)
C15A—Fe1—C14A—C13A	−119.2 (4)	C13B—Fe2—C14B—C15B	−119.2 (4)
C25A—Fe1—C14A—C13A	156.5 (3)	C22B—Fe2—C14B—C15B	−168.2 (4)
C21A—Fe1—C14A—C13A	−169.0 (4)	C11B—Fe2—C14B—C15B	−37.8 (2)
C24A—Fe1—C14A—C13A	113.9 (3)	C23B—Fe2—C14B—C13B	−81.0 (3)
C23A—Fe1—C14A—C13A	73.4 (3)	C21B—Fe2—C14B—C13B	167.0 (7)
C22A—Fe1—C14A—C13A	43.8 (8)	C15B—Fe2—C14B—C13B	119.2 (4)
C12A—Fe1—C14A—C13A	−37.3 (3)	C25B—Fe2—C14B—C13B	−164.4 (3)
C11A—Fe1—C14A—C13A	−81.3 (3)	C12B—Fe2—C14B—C13B	37.3 (3)
C13A—C14A—C15A—C11A	−1.2 (5)	C24B—Fe2—C14B—C13B	−123.9 (3)
Fe1—C14A—C15A—C11A	−60.8 (3)	C22B—Fe2—C14B—C13B	−49.1 (5)
C13A—C14A—C15A—Fe1	59.5 (3)	C11B—Fe2—C14B—C13B	81.4 (3)
C12A—C11A—C15A—C14A	1.7 (5)	C13B—C14B—C15B—C11B	−0.6 (5)
C31A—C11A—C15A—C14A	179.9 (4)	Fe2—C14B—C15B—C11B	59.4 (3)
Fe1—C11A—C15A—C14A	59.8 (3)	C13B—C14B—C15B—Fe2	−59.9 (3)
C12A—C11A—C15A—Fe1	−58.1 (3)	C12B—C11B—C15B—C14B	0.1 (5)
C31A—C11A—C15A—Fe1	120.1 (4)	C31B—C11B—C15B—C14B	−176.2 (4)
C25A—Fe1—C15A—C14A	113.6 (3)	Fe2—C11B—C15B—C14B	−58.8 (3)
C21A—Fe1—C15A—C14A	156.7 (3)	C12B—C11B—C15B—Fe2	59.0 (3)
C13A—Fe1—C15A—C14A	−37.8 (3)	C31B—C11B—C15B—Fe2	−117.4 (4)
C24A—Fe1—C15A—C14A	72.5 (3)	C23B—Fe2—C15B—C14B	−44.7 (5)
C23A—Fe1—C15A—C14A	42.2 (8)	C21B—Fe2—C15B—C14B	−165.0 (3)
C22A—Fe1—C15A—C14A	−170.2 (4)	C25B—Fe2—C15B—C14B	−123.8 (3)
C12A—Fe1—C15A—C14A	−81.3 (3)	C12B—Fe2—C15B—C14B	81.3 (3)
C11A—Fe1—C15A—C14A	−119.4 (4)	C24B—Fe2—C15B—C14B	−80.9 (3)
C14A—Fe1—C15A—C11A	119.4 (4)	C13B—Fe2—C15B—C14B	37.8 (3)
C25A—Fe1—C15A—C11A	−127.0 (3)	C22B—Fe2—C15B—C14B	160.7 (6)
C21A—Fe1—C15A—C11A	−83.9 (3)	C11B—Fe2—C15B—C14B	119.6 (4)
C13A—Fe1—C15A—C11A	81.6 (3)	C14B—Fe2—C15B—C11B	−119.6 (4)
C24A—Fe1—C15A—C11A	−168.1 (3)	C23B—Fe2—C15B—C11B	−164.3 (4)
C23A—Fe1—C15A—C11A	161.6 (7)	C21B—Fe2—C15B—C11B	75.4 (3)
C22A—Fe1—C15A—C11A	−50.8 (5)	C25B—Fe2—C15B—C11B	116.6 (3)
C12A—Fe1—C15A—C11A	38.1 (2)	C12B—Fe2—C15B—C11B	−38.4 (3)
C15A—Fe1—C21A—C22A	157.8 (3)	C24B—Fe2—C15B—C11B	159.5 (3)
C14A—Fe1—C21A—C22A	−167.4 (4)	C13B—Fe2—C15B—C11B	−81.9 (3)
C25A—Fe1—C21A—C22A	−118.1 (4)	C22B—Fe2—C15B—C11B	41.1 (8)
C13A—Fe1—C21A—C22A	41.3 (8)	C14B—Fe2—C21B—C22B	156.3 (6)
C24A—Fe1—C21A—C22A	−80.9 (3)	C23B—Fe2—C21B—C22B	37.3 (3)
C23A—Fe1—C21A—C22A	−36.7 (3)	C15B—Fe2—C21B—C22B	−166.3 (3)
C12A—Fe1—C21A—C22A	73.6 (4)	C25B—Fe2—C21B—C22B	119.6 (4)
C11A—Fe1—C21A—C22A	114.6 (3)	C12B—Fe2—C21B—C22B	−80.5 (3)
C15A—Fe1—C21A—C25A	−84.1 (3)	C24B—Fe2—C21B—C22B	81.6 (3)
C14A—Fe1—C21A—C25A	−49.3 (6)	C13B—Fe2—C21B—C22B	−43.9 (6)
C13A—Fe1—C21A—C25A	159.3 (7)	C11B—Fe2—C21B—C22B	−123.9 (3)
C24A—Fe1—C21A—C25A	37.2 (3)	C14B—Fe2—C21B—C25B	36.7 (9)
C23A—Fe1—C21A—C25A	81.3 (3)	C23B—Fe2—C21B—C25B	−82.4 (3)
C22A—Fe1—C21A—C25A	118.1 (4)	C15B—Fe2—C21B—C25B	74.1 (4)
C12A—Fe1—C21A—C25A	−168.3 (3)	C12B—Fe2—C21B—C25B	159.9 (3)
C11A—Fe1—C21A—C25A	−127.4 (3)	C24B—Fe2—C21B—C25B	−38.0 (3)
C25A—C21A—C22A—C23A	−0.8 (5)	C13B—Fe2—C21B—C25B	−163.5 (4)
Fe1—C21A—C22A—C23A	59.1 (3)	C22B—Fe2—C21B—C25B	−119.6 (4)
C25A—C21A—C22A—Fe1	−60.0 (3)	C11B—Fe2—C21B—C25B	116.4 (3)
C15A—Fe1—C22A—C23A	−167.7 (4)	C25B—C21B—C22B—C23B	0.8 (6)
C14A—Fe1—C22A—C23A	37.6 (9)	Fe2—C21B—C22B—C23B	−58.7 (3)
C25A—Fe1—C22A—C23A	−81.6 (3)	C25B—C21B—C22B—Fe2	59.5 (3)
C21A—Fe1—C22A—C23A	−120.2 (4)	C14B—Fe2—C22B—C23B	−45.7 (5)
C13A—Fe1—C22A—C23A	72.3 (4)	C21B—Fe2—C22B—C23B	119.7 (4)
C24A—Fe1—C22A—C23A	−37.7 (3)	C15B—Fe2—C22B—C23B	163.5 (6)
C12A—Fe1—C22A—C23A	113.3 (3)	C25B—Fe2—C22B—C23B	82.2 (3)
C11A—Fe1—C22A—C23A	156.7 (3)	C12B—Fe2—C22B—C23B	−122.6 (3)
C15A—Fe1—C22A—C21A	−47.5 (5)	C24B—Fe2—C22B—C23B	38.4 (3)
C14A—Fe1—C22A—C21A	157.8 (7)	C13B—Fe2—C22B—C23B	−80.2 (3)
C25A—Fe1—C22A—C21A	38.5 (3)	C11B—Fe2—C22B—C23B	−164.2 (3)
C13A—Fe1—C22A—C21A	−167.5 (3)	C14B—Fe2—C22B—C21B	−165.4 (4)
C24A—Fe1—C22A—C21A	82.5 (3)	C23B—Fe2—C22B—C21B	−119.7 (4)
C23A—Fe1—C22A—C21A	120.2 (4)	C15B—Fe2—C22B—C21B	43.8 (8)
C12A—Fe1—C22A—C21A	−126.6 (3)	C25B—Fe2—C22B—C21B	−37.4 (3)
C11A—Fe1—C22A—C21A	−83.2 (3)	C12B—Fe2—C22B—C21B	117.7 (3)
C21A—C22A—C23A—C24A	0.2 (6)	C24B—Fe2—C22B—C21B	−81.3 (3)
Fe1—C22A—C23A—C24A	58.9 (3)	C13B—Fe2—C22B—C21B	160.2 (3)
C21A—C22A—C23A—Fe1	−58.8 (3)	C11B—Fe2—C22B—C21B	76.2 (3)
C15A—Fe1—C23A—C22A	158.0 (6)	C21B—C22B—C23B—C24B	−1.4 (5)
C14A—Fe1—C23A—C22A	−168.4 (3)	Fe2—C22B—C23B—C24B	−60.1 (3)
C25A—Fe1—C23A—C22A	81.4 (3)	C21B—C22B—C23B—Fe2	58.7 (3)
C21A—Fe1—C23A—C22A	37.2 (3)	C14B—Fe2—C23B—C22B	158.8 (3)
C13A—Fe1—C23A—C22A	−127.2 (3)	C21B—Fe2—C23B—C22B	−37.4 (3)
C24A—Fe1—C23A—C22A	119.9 (4)	C15B—Fe2—C23B—C22B	−169.6 (4)
C12A—Fe1—C23A—C22A	−84.5 (3)	C25B—Fe2—C23B—C22B	−81.1 (3)
C11A—Fe1—C23A—C22A	−48.8 (5)	C12B—Fe2—C23B—C22B	76.3 (3)
C15A—Fe1—C23A—C24A	38.1 (8)	C24B—Fe2—C23B—C22B	−118.5 (4)
C14A—Fe1—C23A—C24A	71.7 (3)	C13B—Fe2—C23B—C22B	116.6 (3)
C25A—Fe1—C23A—C24A	−38.5 (3)	C11B—Fe2—C23B—C22B	45.9 (7)
C21A—Fe1—C23A—C24A	−82.7 (3)	C14B—Fe2—C23B—C24B	−82.7 (3)
C13A—Fe1—C23A—C24A	112.9 (3)	C21B—Fe2—C23B—C24B	81.1 (3)
C22A—Fe1—C23A—C24A	−119.9 (4)	C15B—Fe2—C23B—C24B	−51.1 (6)
C12A—Fe1—C23A—C24A	155.6 (3)	C25B—Fe2—C23B—C24B	37.4 (3)
C11A—Fe1—C23A—C24A	−168.7 (4)	C12B—Fe2—C23B—C24B	−165.2 (3)
C22A—C23A—C24A—C25A	0.6 (5)	C13B—Fe2—C23B—C24B	−124.9 (3)
Fe1—C23A—C24A—C25A	59.9 (3)	C22B—Fe2—C23B—C24B	118.5 (4)
C22A—C23A—C24A—Fe1	−59.3 (3)	C11B—Fe2—C23B—C24B	164.4 (6)
C15A—Fe1—C24A—C25A	73.7 (3)	C22B—C23B—C24B—C25B	1.4 (5)
C14A—Fe1—C24A—C25A	114.4 (3)	Fe2—C23B—C24B—C25B	−59.1 (3)
C21A—Fe1—C24A—C25A	−37.4 (3)	C22B—C23B—C24B—Fe2	60.5 (3)
C13A—Fe1—C24A—C25A	157.2 (3)	C14B—Fe2—C24B—C25B	−125.8 (3)
C23A—Fe1—C24A—C25A	−117.7 (4)	C23B—Fe2—C24B—C25B	119.4 (4)
C22A—Fe1—C24A—C25A	−81.0 (3)	C21B—Fe2—C24B—C25B	38.0 (3)
C12A—Fe1—C24A—C25A	−169.4 (4)	C15B—Fe2—C24B—C25B	−83.3 (3)
C11A—Fe1—C24A—C25A	41.9 (8)	C12B—Fe2—C24B—C25B	161.6 (5)
C15A—Fe1—C24A—C23A	−168.6 (3)	C13B—Fe2—C24B—C25B	−167.1 (3)
C14A—Fe1—C24A—C23A	−127.9 (3)	C22B—Fe2—C24B—C25B	81.7 (3)
C25A—Fe1—C24A—C23A	117.7 (4)	C11B—Fe2—C24B—C25B	−48.9 (6)
C21A—Fe1—C24A—C23A	80.4 (3)	C14B—Fe2—C24B—C23B	114.8 (3)
C13A—Fe1—C24A—C23A	−85.1 (3)	C21B—Fe2—C24B—C23B	−81.5 (3)
C22A—Fe1—C24A—C23A	36.8 (3)	C15B—Fe2—C24B—C23B	157.3 (3)
C12A—Fe1—C24A—C23A	−51.7 (5)	C25B—Fe2—C24B—C23B	−119.4 (4)
C11A—Fe1—C24A—C23A	159.7 (6)	C12B—Fe2—C24B—C23B	42.2 (7)
C23A—C24A—C25A—C21A	−1.1 (5)	C13B—Fe2—C24B—C23B	73.5 (3)
Fe1—C24A—C25A—C21A	58.9 (3)	C22B—Fe2—C24B—C23B	−37.7 (3)
C23A—C24A—C25A—Fe1	−60.0 (3)	C11B—Fe2—C24B—C23B	−168.3 (4)
C22A—C21A—C25A—C24A	1.2 (5)	C22B—C21B—C25B—C24B	0.0 (6)
Fe1—C21A—C25A—C24A	−59.1 (3)	Fe2—C21B—C25B—C24B	59.8 (3)
C22A—C21A—C25A—Fe1	60.3 (3)	C22B—C21B—C25B—Fe2	−59.8 (3)
C15A—Fe1—C25A—C24A	−126.1 (3)	C23B—C24B—C25B—C21B	−0.9 (5)
C14A—Fe1—C25A—C24A	−83.1 (3)	Fe2—C24B—C25B—C21B	−59.7 (3)
C21A—Fe1—C25A—C24A	120.0 (4)	C23B—C24B—C25B—Fe2	58.8 (3)
C13A—Fe1—C25A—C24A	−48.3 (5)	C14B—Fe2—C25B—C21B	−168.2 (3)
C23A—Fe1—C25A—C24A	38.7 (3)	C23B—Fe2—C25B—C21B	80.8 (3)
C22A—Fe1—C25A—C24A	81.9 (3)	C15B—Fe2—C25B—C21B	−126.1 (3)
C12A—Fe1—C25A—C24A	160.1 (7)	C12B—Fe2—C25B—C21B	−47.0 (6)
C11A—Fe1—C25A—C24A	−167.5 (3)	C24B—Fe2—C25B—C21B	118.6 (4)
C15A—Fe1—C25A—C21A	113.8 (3)	C13B—Fe2—C25B—C21B	156.2 (6)
C14A—Fe1—C25A—C21A	156.9 (3)	C22B—Fe2—C25B—C21B	37.3 (3)
C13A—Fe1—C25A—C21A	−168.4 (4)	C11B—Fe2—C25B—C21B	−82.5 (3)
C24A—Fe1—C25A—C21A	−120.0 (4)	C14B—Fe2—C25B—C24B	73.2 (3)
C23A—Fe1—C25A—C21A	−81.4 (3)	C23B—Fe2—C25B—C24B	−37.8 (3)
C22A—Fe1—C25A—C21A	−38.2 (3)	C21B—Fe2—C25B—C24B	−118.6 (4)
C12A—Fe1—C25A—C21A	40.1 (9)	C15B—Fe2—C25B—C24B	115.3 (3)
C11A—Fe1—C25A—C21A	72.5 (3)	C12B—Fe2—C25B—C24B	−165.7 (4)
C15A—C11A—C31A—C32A	45.1 (6)	C13B—Fe2—C25B—C24B	37.6 (8)
C12A—C11A—C31A—C32A	−137.1 (4)	C22B—Fe2—C25B—C24B	−81.3 (3)
Fe1—C11A—C31A—C32A	133.5 (4)	C11B—Fe2—C25B—C24B	158.9 (3)
C15A—C11A—C31A—C36A	−132.9 (4)	C12B—C11B—C31B—C36B	−142.6 (5)
C12A—C11A—C31A—C36A	44.8 (6)	C15B—C11B—C31B—C36B	32.9 (6)
Fe1—C11A—C31A—C36A	−44.5 (5)	Fe2—C11B—C31B—C36B	−53.6 (5)
C36A—C31A—C32A—C33A	2.2 (6)	C12B—C11B—C31B—C32B	35.2 (7)
C11A—C31A—C32A—C33A	−175.8 (4)	C15B—C11B—C31B—C32B	−149.3 (4)
C36A—C31A—C32A—C1	174.0 (4)	Fe2—C11B—C31B—C32B	124.2 (4)
C11A—C31A—C32A—C1	−4.1 (6)	C36B—C31B—C32B—C33B	3.2 (6)
O1—C1—C32A—C31A	−119.4 (5)	C11B—C31B—C32B—C33B	−174.6 (4)
N1—C1—C32A—C31A	60.1 (6)	C36B—C31B—C32B—C2	−169.0 (4)
O1—C1—C32A—C33A	52.7 (6)	C11B—C31B—C32B—C2	13.1 (6)
N1—C1—C32A—C33A	−127.8 (4)	O2—C2—C32B—C33B	−126.6 (4)
C31A—C32A—C33A—C34A	−0.4 (6)	N1—C2—C32B—C33B	49.4 (5)
C1—C32A—C33A—C34A	−173.0 (4)	O2—C2—C32B—C31B	46.0 (6)
C32A—C33A—C34A—C35A	−1.2 (6)	N1—C2—C32B—C31B	−138.1 (4)
C33A—C34A—C35A—C36A	1.0 (6)	C31B—C32B—C33B—C34B	−2.5 (6)
C34A—C35A—C36A—C31A	0.9 (6)	C2—C32B—C33B—C34B	170.2 (4)
C32A—C31A—C36A—C35A	−2.5 (6)	C32B—C33B—C34B—C35B	0.4 (6)
C11A—C31A—C36A—C35A	175.6 (4)	C33B—C34B—C35B—C36B	0.9 (6)
C1—N1—C3—N8	−130.6 (4)	C34B—C35B—C36B—C31B	0.0 (7)
C2—N1—C3—N8	31.6 (5)	C32B—C31B—C36B—C35B	−2.0 (6)
C1—N1—C3—C4	49.7 (5)	C11B—C31B—C36B—C35B	176.0 (4)

### Hydrogen-bond geometry (Å, °)

**Table:**

*D*—H···*A*	*D*—H	H···*A*	*D*···*A*	*D*—H···*A*
C9—H9B···O1^i^	0.98	2.36	3.188 (6)	142
C13B—H13B···O2^ii^	0.95	2.58	3.510 (6)	167
C33B—H33B···Cg1	0.95	2.58	3.387 (5)	144
C35A—H35A···Cg2^iii^	0.95	2.61	3.489 (6)	154

Symmetry codes: (i) −*x*+2, −*y*, −*z*+2; (ii) −*x*+2, −*y*+1, −*z*+1;  (iii) −*x*+1, −*y*+1, −*z*+2.
